# Open Transcatheter Multivalve Replacement in Degenerated Valve Prostheses in High-Risk Patients with Endocarditis

**DOI:** 10.21470/1678-9741-2020-0394

**Published:** 2021

**Authors:** Alina Zubarevich, Konstantin Zhigalov, Arian Arjomandi Rad, Robert Vardanyan, Daniel Wendt, Bastian Schmack, Arjang Ruhparwar, Alexander Weymann

**Affiliations:** 1 Department of Thoracic and Cardiovascular Surgery, West German Heart and Vascular Center, University of Duisburg-Essen, Essen, Germany.; 2 Department of Medicine, Faculty of Medicine, Imperial College London, London, UK.

**Keywords:** Transcatheter Valve Replacement, Mitral Valve, Heart Valve Prosthesis, Endocarditis.

## Abstract

Multivalve redo procedures carry a high surgical risk. We describe an alternative surgical treatment for patients presenting with severely degenerated aortic and mitral valve prostheses who have to undergo open surgery due to endocarditis. Open transcatheter multivalve implantation is a feasible bailout strategy in high-risk patients to save cross-clamp and procedural times to reduce morbidity and mortality.

**Table:** 

Abbreviations, acronyms & symbols				
AL	= Anterior leaflet		LVF	= Left ventricular function
AV	= Aortic valve		MV	= Mitral valve
BMI	= Body mass index		RA	= Right atrium
CPB	= Cardiopulmonary bypass		RV	= Right ventricle
CT	= Computed tomography		PET	= Positron emission tomography
IE	= Infective endocarditis		TAVI	= Transcatheter aortic valve implantation
LA	= Left atrium		TV	= Tricuspid valve

## INTRODUCTION

Surgical mitral and aortic valve replacement in younger patients carries higher odds of reoperation due to valve degeneration over the course of time. As combined redo valve procedures are known to involve a high surgical risk, there is a constant discussion about an alternative surgical treatment option with lower mortality and morbidity rates.

We describe an alternative surgical treatment for patients presenting with severely degenerated aortic and mitral valve prostheses who have to undergo open surgery due to endocarditis.

### Scenario

A 61-year-old female patient presenting with progressive dyspnea and cardiac decompensation due to severe stenosis of degenerated mitral and aortic valve (MV, AV) prostheses, and concomitant infective endocarditis (IE) of the tricuspid valve (TV). The patient underwent a MV and AV replacement with a 29-mm Hancock II and a 21-mm Perimount Magna prostheses, respectively, performed via median sternotomy, two years ago. Upon admission to the clinic, the patient underwent coronary angiography, excluding coronary arterial disease. The echocardiogram showed good left ventricular function (LVF), severe stenotic AV and MV prostheses and infective vegetation on the TV. The right ventricle (RV) was enlarged with a slightly impaired function and a systolic pulmonary arterial pressure of 80 mmHg. PET-CT scan confirmed IE of the TV. In the meantime, the blood cultures showed infection by *Staphylococcus capitis* and *Staphylococcus epidermidis.* Antibiotic therapy was planned for six weeks postoperatively. Further medical history of the patient included insulin-dependent diabetes mellitus with diabetic nephropathy and dialysis, hypertension, severe chronic obstructive pulmonary disease, and severe obesity with a BMI of 41.1 kg/m^2^. Logistic EuroSCORE was estimated at over 80%. The Heart Team decided for transcatheter implantation of the MV and AV combined with a TV procedure on the beating heart to save procedural and cross-clamp times.

## TECHNIQUE

After median re-sternotomy and going through severe adhesions after chest reconstruction, a cardiopulmonary bypass (CPB) was established via cannulation of the ascending aorta and both caval veins. The technique was as follows:

- TV procedure was performed on the beating heart. After gaining access to the right atrium (RA), a 5-mm round structure on the anterior leaflet (AL) of the TV was noticed. AL was radically excised, and a valve bicuspidalisation was performed. A TV annuloplasty was performed with a 31-mm Duran band (Medtronic, Minneapolis, MN). The saline test revealed no significant TV regurgitation. RA was closed with a Prolene 4-0 double running suture.
- The cardioplegic arrest was established via antegrade infusion of cold Custodiol solution (Köhler Chemie GmbH, Bensheim, Germany). After opening the left atrium (LA) and examining the degenerated MV prosthesis (Figures 1A and B), no signs of IE were found. The leaflets of the MV prosthesis were radically excised (Figures 1C and D). Aortotomy was performed 2 cm above the calcified AV prosthesis, and the prosthesis leaflets were also removed.
  **Fig. 1:**
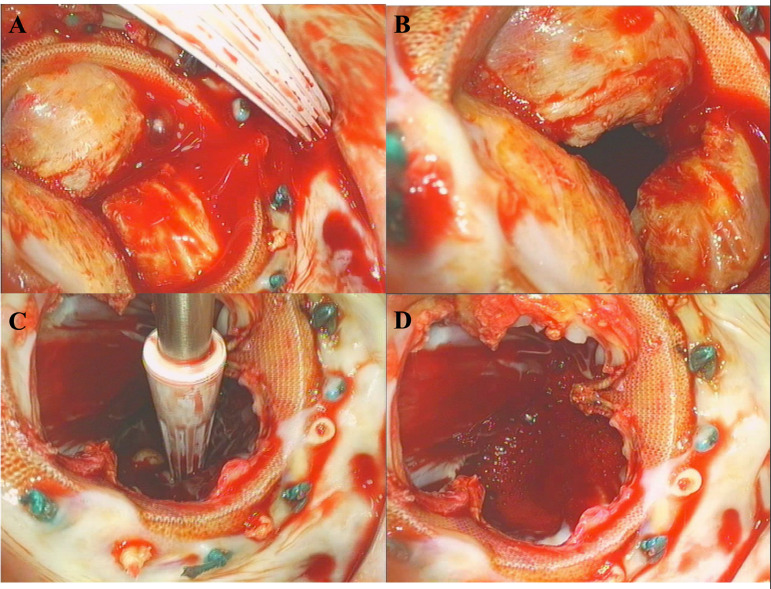
Deteriorated mitral valve prosthesis in situ. A) calcified mitral valve prosthesis; B) coaptation defect; C-D) excised leaflets of the deteriorated mitral valve prosthesis.
- Subsequently, the deployment at the nominal volume of a 26-mm Sapien Ultra (Edwards Lifesciences, Irvine, USA) into the MV prosthesis was performed under direct vision (Figures 2 A and B). We noticed a satisfactory expansion of the prosthesis (Figures 2 C and D), thus no further post-dilatation was necessary.
  **Fig. 2:**
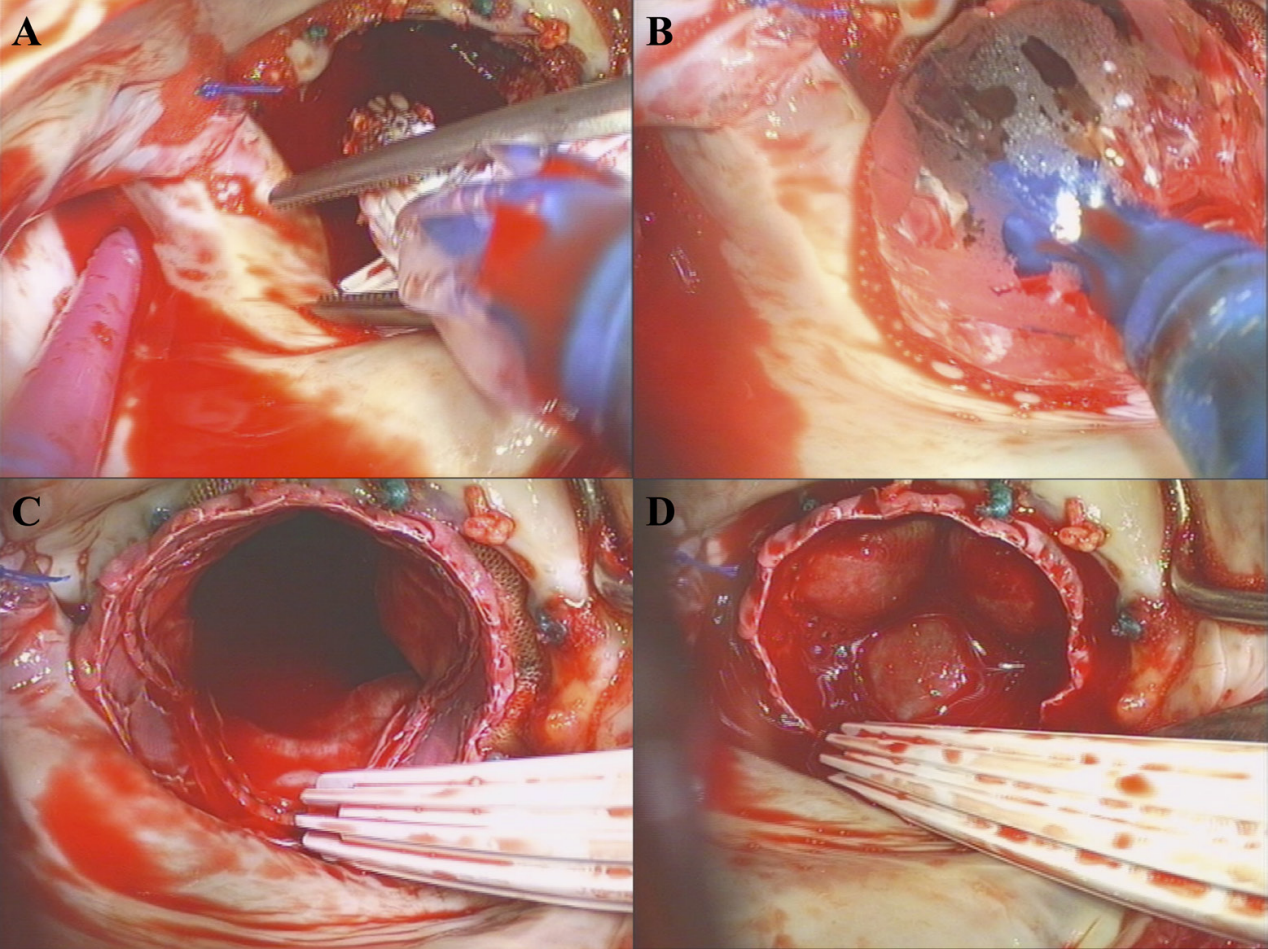
Open transcatheter valve implantation. A) positioning of the TAVI system in the mitral valve annulus; B) balloon dilatation of the TAVI prosthesis; C) TAVI prosthesis in mitral position, left atrial view; D) competent TAVI prosthesis in mitral position, left atrial view.
- A 23-mm Sapien Ultra (Edwards Lifesciences, Irvine, USA) transcatheter valve was deployed under direct vision into the degenerated AV prosthesis. We assured a correct position of the prosthesis. The weaning from CPB after 24 minutes of cross-clamping was uneventful.
- The intraoperative transesophageal echocardiography showed satisfactory function of all three corrected valves with no paravalvular leakage of the transcatheter prostheses and a mild TV regurgitation.
- After thorough hemostasis, an extensive chest closure with a skin mesh graft on the caudal end of the sternotomy was performed. A stable chest closure has been achieved.

### Postoperative Course

After the surgical procedure, the patient was transferred from the operating room to the intensive care unit with low-dose catecholamines (noradrenaline 0.18 µg/kg/min and adrenaline 0.03 µg/kg/min). The patient was extubated 26 hours following the procedure and further postoperative course was uneventful.

## DISCUSSION

Choosing a biological valve prosthesis in younger patients carries the risk of rapid prosthesis degeneration and, eventually, high-risk re-operation^[1]^. In general, the guidelines do not recommend biological valve prostheses in patients under 65 years old^[2]^. Nevertheless, there are controversial cases in which different concepts are part of an ongoing debate, such as in patients on chronic dialysis^[3]^. In the aforementioned case, a 59-year-old patient was treated with biological AV and MV prostheses and unfortunately suffered a rapid degeneration of both valves two years postoperatively. Due to IE of the TV, the patient was not eligible for the regular transcatheter procedure. Furthermore, her logistic EuroSCORE of over 80% indicated an excessively high mortality risk for a conventional redo surgery. Operative and cross-clamp times are known to be predictive factors for postoperative morbidity and mortality^[4]^. Nevertheless, it is possible to shorten these times with sutureless valve prostheses^[5,6]^. We had already described the implantation of a Perceval prosthesis in mitral position^[7]^ in a high-risk setting. Open aortic transcatheter valve implantation is a feasible bailout option in high-risk redo procedures to avoid a redo aortic root replacement^[8]^. In the following case, we successfully managed to implant two transcatheter valves into the deteriorated prostheses under direct vision. Thanks to the TV procedure being performed on the beating heart, we were able to reduce the clamping time to 24 minutes.

## CONCLUSION

The combination of an open-heart surgery with CPB and transcatheter AV and MV implantation significantly reduces the surgical risk compared to a conventional triple-valve redo procedure. It also facilitates avoiding major complications and technical difficulties, which would have been inevitably faced in a conventional redo surgery.

**Table:** 

Authors' roles & responsibilities	
AZ	Substantial contributions to the conception or design of the work; or the acquisition, analysis, or interpretation of data for the work; drafting the work or revising it critically for important intellectual content; agreement to be accountable for all aspects of the work in ensuring that questions related to the accuracy or integrity of any part of the work are appropriately investigated and resolved; final approval of the version to be published
KZ	Substantial contributions to the conception or design of the work; or the acquisition, analysis, or interpretation of data for the work; drafting the work or revising it critically for important intellectual content; agreement to be accountable for all aspects of the work in ensuring that questions related to the accuracy or integrity of any part of the work are appropriately investigated and resolved; final approval of the version to be published
AAR	Substantial contributions to the conception; agreement to be accountable for all aspects of the work; final approval of the version to be published
RV	Substantial contributions to the conception; agreement to be accountable for all aspects of the work; final approval of the version to be published
DW	Substantial contributions to the conception; agreement to be accountable for all aspects of the work; final approval of the version to be published
BS	Substantial contributions to the conception; agreement to be accountable for all aspects of the work; final approval of the version to be published
AR	Substantial contributions to the conception; agreement to be accountable for all aspects of the work; final approval of the version to be published
AW	Agreement to be accountable for all aspects of the work in ensuring that questions related to the accuracy or integrity of any part of the work are appropriately investigated and resolved; final approval of the version to be published
